# Association of IgG co-deposition with serum levels of galactose-deficient IgA1 in pediatric IgA nephropathy 

**DOI:** 10.5414/CN107423

**Published:** 2012-09-25

**Authors:** T. Matthew Eison, M. Colleen Hastings, Zina Moldoveanu, John T. Sanders, Lillian Gaber, Patrick D. Walker, Keith K Lau, Bruce A. Julian, Jan Novak, Robert J. Wyatt

**Affiliations:** 1 University of Tennessee Health Science Center and Children’s Foundation Research Center at Le Bonheur Children’s Hospital, Memphis, TN,; 2 University of Alabama at Birmingham, Birmingham, AL,; 3 Sanford Children’s Hospital, Sioux Falls, SD,; 4 Department of Pathology, The Methodist Hospital, Houston, TX,; 5 Nephropathology Associates, Little Rock, AR, USA, and; 6 McMaster University, Hamilton, Ontario, Canada

**Keywords:** IgA nephropathy, galactose-deficient IgA1, glomerular disease, glomerulonephritis

## Abstract

Objective: To determine whether the absence of mesangial IgG deposits is associated with the absence of elevated blood levels of galactose-deficient IgA1 (Gd-IgA1) in pediatric patients with IgA nephropathy (IgAN). Design and methods: Serum Gd-IgA1 levels were determined by ELISA using an *N-*acetylgalactosamine-specific lectin from *Helix aspersa*. Levels of Gd-IgA1 above the 90th percentile for healthy pediatric controls were considered to be elevated. Renal biopsy samples were examined by immunofluorescence for presence and intensity of staining for IgA, IgG, IgM, C3 and C1q and by light microscopy for histological changes. Findings were graded by a single pathologist (L. Gaber) at UTHSC until 2007 and by Nephropath^TM^ (Little Rock, AR, USA) thereafter. Staining for the mesangial deposits was considered negative when intensity was trace or less, and positive at greater intensity. Fisher’s exact-test was used to determine significance of 2 × 2 tables. Results: Serum samples were obtained from 30 patients with IgAN diagnosed before age 18 years. Male : female ratio was 2.3 : 1. Twenty were Caucasian and 10 were African-American. Blood was obtained within 3 months of biopsy (incident cases) for 12, while 18 provided blood > 3 months after biopsy (prevalent cases). Serum Gd-IgA1 level was elevated in 23 (77%) of cases and 20 (67%) had a biopsy positive for IgG. Of those 20 patients, 18 (90%) had an elevated serum Gd-IgA1 level, whereas 5 (50%) of patients with biopsies without IgG had a normal serum Gd-IgA1 level (p = 0.026). Summary: In this small study we found a weak association between the absence of IgG in the biopsy and normal serum Gd-IgA1 level.

## Introduction 

IgA nephropathy (IgAN) is an autoimmune renal disease resulting from aberrant glycosylation of IgA1 in the hinge-region *O*-linked glycans. Some of the carbohydrate side chains lack galactose and thus terminate with *N*-acetylgalactosamine (GalNAc) with or without sialic acid. GalNAc can be recognized by anti-glycan IgG antibodies, resulting in formation of immune complexes either *in situ* in the mesangium or in the circulation that can subsequently deposit in the mesangium [1, 2, 3]. We have previously shown that 75% of pediatric patients with IgAN have elevated serum levels of galactose-deficient IgA1 (Gd-IgA1) [4]. It is not known whether Gd-IgA1 is recognized by cross-reactive antibodies originally induced by a mucosal pathogen, for example, or whether Gd-IgA1 may be inducing these IgG autoantibodies. As many adult patients with IgAN do not have IgG co-deposits [5], we assessed whether pediatric patients with IgAN exhibit an association between presence of IgA deposits and serum Gd-IgA1 level. In theory, such an association would be supportive of the immunogenic character of Gd-IgA1 in IgAN. 

## Subjects and methods 

Serum samples were collected from 30 patients diagnosed with IgAN before age 18 years. The male to female ratio was 2.3 : 1. 20 were Caucasian and 10 were African-American. Blood was obtained within 3 months of biopsy (incident cases) for 12, or more than 3 months from biopsy (prevalent cases) for 18. 

Serum samples were obtained from 97 healthy controls younger than age 18 years. These were comprised of 29 African-American males, 21 African-American females, 28 Caucasian males and 19 Caucasian females. The mean ± SD age for the controls at time of study was 12.6 ± 2.9 years. 

The levels of Gd-IgA1 were determined in serum samples by ELISA using a GalNAc-specific lectin from *Helix aspersa* after removal of terminal sialic acid by neuraminidase treatment [6]. The median serum Gd-IgA1 level for the healthy controls was 260 units/ml, with a range of 81 – 998 units/ml. A serum Gd-IgA1 level was defined as elevated if it was above 500 units/ml, the 90^th^ percentile for the controls. 

Renal biopsies were examined by immunofluorescence (IF) microscopy using fluorochrome-labeled antibodies specific for human IgG, IgA, IgM, C3 and C1q, and were interpreted by a single pathologist (L. Gaber) at UTHSC until 2007 and by Nephropath^TM^ (Little Rock, AR, USA) thereafter. The fluorescence intensity was graded 0, trace, 1, 2 or 3. For the purposes of this study, those evaluated as trace or zero were considered negative and those with intensity of 1, 2 or 3 were grouped as positive. The light-microscopic features of the renal biopsy specimens were graded according to the Oxford classification system by review of available slides by a single renal pathologist (P. Walker) [7]. 

Statistical analysis: Fisher’s exact-test was used to determine significance of 2 × 2 tables. The D’Agnostino and Pearson omnibus normality test was used to determine whether the serum Gd-IgA1 levels for the controls fit a normal distribution. The Mann-Whitney U-test was used to determine differences for continuous variables for renal biopsies positive for IgG vs. those without IgG deposits. 

## Results 

**Figure 1.**
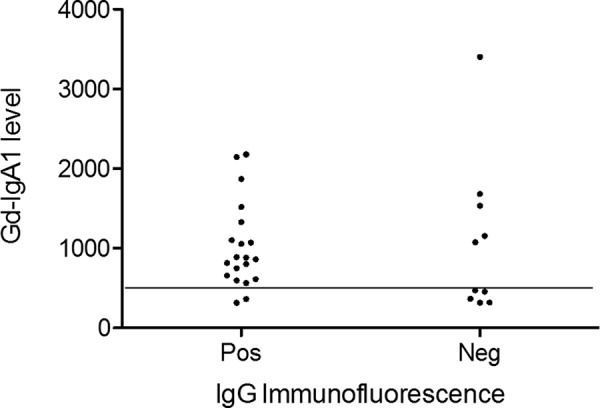
This figure shows the association between presence (Pos) or absence (Neg) of immunofluorescence (IF) for IgG and serum Gd-IgA1 level. Of 7 patients with normal serum Gd-IgA1 levels, 5 had negative IF for IgG (p = 0.026, Fisher’s exact test, two-tailed).

The relationship between the mesangial IF staining for IgG and serum Gd-IgA1 level is shown in Figure 1. No IgG was detected in the biopsy specimens for 10 (33%) of the patients with IgAN. Serum Gd-IgA1 level was elevated in 23 (77%) of the patients with IgAN. Of the 20 patients with IgG mesangial deposits, 18 (90%) had an elevated serum Gd-IgA1 level; for the 10 patients without mesangial staining for IgG, 5 (50%) had a normal Gd-IgA1 level (p = 0.026). C3 was present in 28 biopsies. The 2 patients without C3 had no IgG deposits and a normal Gd-IgA1 level. Absence of mesangial IgG was found in 8 of 22 (36%) of biopsies examined by Dr. Gaber and 2 of the 8 specimens (25%) examined by Nephropath™. The median Gd-IgA1 level was not different with respect to presence or absence of IgG. 

The detailed IF findings in the renal biopsy specimens, the serum levels of Gd-IgA1, and clinical and demographic features for the 10 subjects without IgG mesangial deposits are shown in Table 1. There was no feature other than the absence of IgG in the mesangial deposits that distinguished this group from the 20 patients with IgAN with IgG mesangial deposits. Seven of the 10 subjects without IgG did not have IgM deposits, and thus IgA was the only immunoglobulin detected in the mesangial deposits by IF. Median serum Gd-IgA1 levels for subjects with and without IgG deposits were similar. 

The subgroup of patients with IgG staining did not differ significantly from the subgroup without IgG staining in respect to being incident or prevalent cases for timing of the measurement of serum Gd-IgA1 (Table 2). There was no significant difference between these subgroups for any other feature, such as gender, race, minimal-glomerular-change histology, or presentation with macroscopic hematuria. While the only 3 patients who progressed to chronic kidney disease Stage 4/5 had IgG deposits, the finding was not statistically significant. 

## Discussion 

We reviewed several pediatric studies that reported IF findings in renal biopsy specimens from at least 19 patients with IgAN (Table 3) [8, 9, 10, 11, 12, 13, 14, 15, 16]. There was considerable variation among the studies with respect to the detection of IgG, ranging from 32% to 86%. The cumulative frequency of mesangial IgG was 55% in patients from the United States and Europe vs. 37% in patients in Asia. For the studies summarized in Table 3, the percentages of biopsy specimens from children with IgG staining were similar to those from adult patients with IgAN [5, 17]. 

A multivariate analysis of 27 adult IgAN patients showed that mesangial IgG deposition was associated with disease progression [18]. Recent examination of combined pediatric and adult outcome data from the Oxford Classification study using Cox regression analysis showed a trend toward poorer renal survival for IgG-positive patients as compared to IgG-negative patients [19]. If these studies are confirmed, IgG deposits would become an additional biopsy marker of poor clinical outcome. The findings from our small cohort do not support these observations, as the 8 patients with minimal glomerular changes (Oxford M0 E0 S0 T0) did not differ from those with more severe glomerular lesions on light microscopy with respect to absence of IgG. The percentage of biopsies with Oxford M0 S0 E0 I0 is a good indicator of the degree of mild disease in a given cohort. However, our study with only 30 subjects with IgAN is clearly underpowered to address this question. A more extensive analysis, such as the one done in the original Oxford Classification cohort, would be needed to detect associations between specific histologic lesions and serum Gd-IgA1 levels. 

It is not known whether Gd-IgA1 in IgAN patients can induce IgG autoantibodies specific for the aberrantly glycosylated IgA1 hinge-region glycopeptides. Alternatively, Gd-IgA1 can be recognized by the IgG antibodies originally induced by mucosal microorganisms that cross-react with Gd-IgA1. In this cohort of pediatric patients with IgAN, we observed an association between mesangial IgG deposits and elevated serum Gd-IgA1 level. This finding can thus be interpreted as supporting the immunogenic character of Gd-IgA1 in this group of IgAN patients. It is not clear how to explain the absence of IgG in the mesangial deposits of some patients with IgAN. One possibility is that Gd-IgA1 is recognized by IgA1 antibodies in this group of patients [3] and that these antibodies would be primarily induced by mucosal microorganisms and be cross-reactive with Gd-IgA1. Moreover, other mechanisms may be involved in the formation of IgA deposits without IgG and or IgM co-deposits. 

In a closely related disease, Henoch-Schönlein purpura, the patients with nephritis have IgA-IgA and IgA-IgG circulating immune complexes, whereas patients without nephritis have only IgA-IgA circulating immune complexes [20]. However, in IgAN it is not well understood how the composition of circulating immune complexes affects the disease presentation, severity, and/or progression. 

In summary, in a small cohort we found that pediatric patients with IgAN who do not have mesangial IgG deposits are more likely than those with IgG deposits to have a normal serum Gd-IgA1 level. In this subset of patients, other mechanism(s), not yet elucidated, may drive the pathogenesis of the disease. 

## Acknowledgments 

This study was supported by a generous gift to the UTHSC Pediatric Nephrology Research Fund by Don and Anna Waite and grants from the LeBonheur Children’s Hospital, and the IgA Nephropathy Foundation of America. Robert J. Wyatt, Jan Novak, Bruce A. Julian, and Zina Moldoveanu were supported by grant number DK082753 from the National Institute of Diabetes and Digestive and Kidney Diseases (NIDDK). John T. Sanders was supported by a fellowship training award from the National Kidney Foundation. The authors also acknowledge other grants from NIDDK supporting their research of IgAN: DK078244, DK080301, DK075868, DK083663, DK071802, DK077279, and GM098539. The contents of this publication are solely the responsibility of the authors and do not necessarily represent the official view of NCRR, NIDDK, or NIH. 

**Table 1.** Clinical and demographic features, serum Gd-IgA1 level, immunofluorescence pattern, and Oxford histologic score for patients without mesangial IgG.

Patient	Age at biopsy (y)	Incident/ Prevalent	Race	Gender	Macroscopic hematuria at clinical onset	Gd-IgA1 (units/ml)	IgA	IgM	C3	C1q	M	E	S	T
1	11.2	incident	Caucasian	female	yes	1,076	3+	neg	1 – 2+	neg	1	0	1	0
2	13.4	incident	Caucasian	male	yes	1,535	3+	neg	1+	neg	1	0	0	0
3	12.5	incident	Caucasian	female	yes	1,155	3+	3+	3+	neg	1	0	1	1
4	8.8	incident	African American	male	yes	470	3+	neg	2+	neg	1	0	0	0
5	15.0	incident	Caucasian	male	yes	364	2 – 3+	trace	2-3+	neg	1	1	1	0
6	10.7	prevalent	African American	male	yes	1,682	3+	1+	1+	neg	1	0	0	0
7	11.6	prevalent	Caucasian	female	no	317	3+	neg	3+	neg	0	0	1	0
8	13.4	prevalent	African American	male	no	315	1 – 2+	neg	1-2+	neg	0	0	0	0
9	12.9	prevalent	African American	male	no	456	2+	1+	1+	neg	0	0	0	0
10	15.0	incident	African American	female	yes	3,401	3+	neg	3+	neg	1	0	0	1

Columns M, E, S and T provide Oxford classification scores for mesangial proliferation, endocapillary proliferation, segmental glomerulosclerosis, and tubulointerstitial changes, respectively. Subject 10 is the only patient in this table to have acute cellular crescent formation (100% of 3 glomeruli).

**Table 2.** Clinical and demographic features of patients with and without mesangial IgG deposits.

	IgG positive n = 20	IgG negative n = 10	p value
Incident cases	6	6	0.14*
Prevalent cases	14	4	
Male	16	5	0.12*
Female	4	5	
Caucasian	15	5	0.40*
African-American	5	5	
Age at biopsy	10.8 ± 3.7	12.5 ± 1.9	
Macroscopic hematuria at onset	18	6	0.14*
Years after biopsy for Gd-IgA1 level	1.9 ± 2.2	0.8 ± 1.0	0.13**
Follow-up after biopsy, y, median (range)	4.4 (0.1 – 9.9)	3.0 (0.9 – 6.6)	0.17**
CKD 1/2 at last follow-up	17	10	
CKD 5 at last follow-up	3 (14%)	0	0.53*
Oxford mesangial score = 1	12 (63%)^#^	7 (70%)	1.00*
Oxford endocapillary proliferation score = 1	5 (26%)^#^	1 (10%)	0.63*
Oxford segmental glomerulosclerosis score = 1	4 (21%)^#^	3 (30%)	0.66*
Oxford tubulointerstitial score = 1	0 (0%)^#^	2 (20%)	0.11*
Oxford M0, E0, S0, T0	6 (32%)^#^	2 (20%)	0.67*
Median serum Gd-IgA1 level	773 U/dl	872 U/dl	0.69**

*Fisher’s exact-test; **Mann-Whitney U-test; ^#^one patient had no slides available for review (denominator = 29).

**Table 3.** Immunofluorescence pattern of mesangial deposits in pediatric studies of IgA nephropathy.

Study	Region	# Patients	IgG+	IgM+	C3+	C1q+
Michalk et al., 1980* [13]	Germany	19*	70%	15%	85%	13%**
Kher et al., 1983 [10]	Cleveland, OH, USA	21	86%	62%	86%	19%
Mina et al., 1985 [14]	Memphis, TN, USA	24	37%	4%	75%	–
Levy et al., 1985 [12]	France	91	60%	–	87%	–
Hattori et al., 1985 [8]	Japan	22	41%	14%	73%	–
Yoshikawa et al., 1988-89 [16]	Japan	258	32%	8%	64%	–
Kim et al., 1988-89 [11]	S. Korea	42	55%	45%	81%	–
Okada et al., 1990 [15]	Japan	61	59%	49%	87%	16%
Hogg et al., 1994 [9]	Southwest region, USA	218	53%	29%	80%	–
Present study - Caucasians	Memphis, TN, USA	20	75%	45%	100%	5%
Present study - African Americans	Memphis, TN, USA	10	50%	70%	90%	10%

*includes second biopsy for one patient; **2 of 15 positive.
